# Long-term survival after intensive care unit discharge in Thailand: a retrospective study

**DOI:** 10.1186/cc13036

**Published:** 2013-10-03

**Authors:** Nantasit Luangasanatip, Maliwan Hongsuwan, Yoel Lubell, Direk Limmathurotsakul, Prapit Teparrukkul, Sirirat Chaowarat, Nicholas P J Day, Nicholas Graves, Ben S Cooper

**Affiliations:** 1 Mahidol-Oxford Tropical Medicine Research Unit, Faculty of Tropical Medicine, Mahidol University, Bangkok, Thailand; 2 School of Public Health, Queensland University of Technology, Brisbane, QLD, Australia; 3 Centre for Tropical Medicine, Nuffield Department of Clinical Medicine, University of Oxford, Oxford, United Kingdom; 4 Department of Tropical Hygiene, Faculty of Tropical Medicine, Mahidol University, Bangkok, Thailand; 5 Department of Medicine, Sappasithiprasong Hospital, Ubon Ratchatani, Thailand; 6 Department of Nursing, Sappasithiprasong Hospital, Ubon Ratchatani, Thailand; 7 Institute of Health and Biomedical Innovation, Queensland University of Technology, Brisbane, QLD, Australia

## Abstract

**Introduction:**

Economic evaluations of interventions in the hospital setting often rely on the estimated long-term impact on patient survival. Estimates of mortality rates and long-term outcomes among patients discharged alive from the intensive care unit (ICU) are lacking from lower- and middle-income countries. This study aimed to assess the long-term survival and life expectancy (LE) amongst post-ICU patients in Thailand, a middle-income country.

**Methods:**

In this retrospective cohort study, data from a regional tertiary hospital in northeast Thailand and the regional death registry were linked and used to assess patient survival time after ICU discharge. Adult ICU patients aged at least 15 years who had been discharged alive from an ICU between 1 January 2004 and 31 December 2005 were included in the study, and the death registry was used to determine deaths occurring in this cohort up to 31^st^ December 2010. These data were used in conjunction with standard mortality life tables to estimate annual mortality and life expectancy.

**Results:**

This analysis included 10,321 ICU patients. During ICU admission, 3,251 patients (31.5%) died. Of 7,070 patients discharged alive, 2,527 (35.7%) were known to have died within the five-year follow-up period, a mortality rate 2.5 times higher than that in the Thai general population (age and sex matched). The mean LE was estimated as 18.3 years compared with 25.2 years in the general population.

**Conclusions:**

Post-ICU patients experienced much higher rates of mortality than members of the general population over the five-year follow-up period, particularly in the first year after discharge. Further work assessing Health Related Quality of Life (HRQOL) in both post-ICU patients and in the general population in developing countries is needed.

## Introduction

Hospital mortality amongst intensive care unit (ICU) patients is high throughout the world, typically ranging from 14 to 44% [1-6]; in Thailand the reported range is between 24 and 40% [5-7]. Interventions to improve the quality of ICU care have the potential to reduce this mortality. Examples of such interventions include development of clinical guidelines [8,9], improvements to infection control practices [10], and appropriate use of medical devices [11].

There is growing interest not just in the effectiveness of such interventions at reducing mortality, but in their cost-effectiveness, and formal economic evaluation is increasingly used to aid decisions about allocation of scarce health care resources in these settings [12]. Such analyses consider both costs of the interventions and the associated health benefits. Outcomes such as the number of life years (LYs) or quality adjusted life years (QALYs) gained or disability adjusted life years (DALYs) averted are commonly used to represent the benefit of particular interventions. However, to estimate the change in LYs caused by preventing a single ICU death, estimates of post-ICU survival are needed. A number of studies have assessed long-term survival (defined as survival for at least one year post-ICU discharge) [2,13-29]. All but one of these studies were conducted in high-income countries and high quality data are lacking from lower and middle income countries [26]. The aim of this study was to quantify the long-term survival of post-ICU patients in Thailand and to estimate life expectancy (LE) in this population.

## Materials and methods

### Setting and facilities

Sappasithiprasong Hospital is a 1,100-bed tertiary referral hospital located in rural northeast Thailand. In 2004 and 2005 it had a catchment of 1.8 million people, predominantly rice farmers and their families. Universal health coverage has been operating in Thailand since 2002, ensuring access to this hospital for the entire population in the catchment area [30]. In 2004, Sappasithiprasong Hospital had 36 general wards and 16 ICUs (4 pediatric and 12 adult), representing 6 ICU beds per 100,000 people. These wards provided care for critically ill patients and patients recovering from major surgery. Adult ICUs comprised four medical ICUs (including one respiratory ICU), two surgical ICUs, two neurosurgical ICUs, one trauma ICU, two coronary care units, and one cardiovascular and thoracic ICU. ICUs contained a median of 8 beds (range 8 to 16) and the mean nurse-to-patient ratio was 1:1.5 (including both registered nurses and practical nurses). All of these ICUs could be defined as Level II open ICUs according to the guidelines from the American College of Critical Care Medicine [31] since there were no intensivists accredited by the Royal College of Physicians of Thailand working at Sappasithiprasong Hospital in 2004. Further details about the ICUs in this hospital have been described elsewhere [32].

### Data

Retrospective patient-level data from January 2004 to December 2005 were obtained from Sappasithiprasong Hospital. Adult patients, aged at least 15 years, who had been admitted to an adult ICU and discharged alive from the ICU between 1 January 2004 and 31 December 2005 were included in this retrospective cohort analysis. For patients who were subsequently readmitted to an ICU during this period, only the time since the end of the first ICU episode was considered. The regional death registry for northeast Thailand from 2004 to 2010 was obtained from the Thai Ministry of Public Health and linked to the patient data using the national identification number (ID). We verified the validity of each patient’s ID number using the checksum digit and cross-checked the name and date of birth between hospital data and the regional death registry to validate the data.

Use of these data was approved by ethical committees from 1) the Faculty of Tropical Medicine, Mahidol University, 2) Sappasithiprasong Hospital, Ubon Ratchatani, and 3) the Ministry of Public Health, Thailand [33]. No patient consent was required as this study was retrospective and did not use patient identifiable data.

Patients with a recorded date of death during the ICU admission period were classified as ICU deaths. It is not uncommon practice in Thailand and other Southeast Asian countries to discharge moribund patients to die at home [33]. We, therefore, also classified deaths occurring within two days of ICU discharge as ICU deaths. Survival time for discharged patients was assessed for five years after hospital discharge. Patients were assumed to be alive if no death was recorded within five years of ICU discharge in the death registry.

### Analysis

The primary outcome was survival time after ICU discharge. A Kaplan-Meier survival curve showing the estimated proportion of post-ICU patients alive at each time point was plotted over the five-year follow-up period. To quantify the potential impact of differential mortality following year five, we fitted an exponential curve to the annual risk of death from years two to five. From year eight onwards, the extrapolated post-ICU mortality differed by less than 1% from that in the general population matched for age and sex. Therefore, mortality from year eight was assumed to be equal to that in the general Thai population which we took from standard mortality life tables [34]. In the base case analysis we assumed that in years six and seven post-discharge the relative risk of death for former ICU patients compared to the general population was the same as that observed in year five (relative risk of 1.35). Since this assumption may underestimate post-ICU survival, we performed a sensitivity analysis in which we assumed that mortality rates in years six and seven post-discharge were the same as those in the general population matched for age and sex (that is, a relative risk of one). The life expectancy (LE) amongst patients discharged from the ICU was taken as the area under the lifetime survival curve. The LE was calculated for the overall ICU population and for each age group.

Survival analysis stratified according to major diagnostic categories for ICU admission from the International Statistical Classification of Diseases, 10^th^ revision (ICD10) [35] was also performed. The diagnostic groups were: a.) Cerebrovascular diseases (ICD10 codes: I60 to I69); b.) Cardiovascular diseases except Cerebrovascular diseases (ICD10 codes: I00 to I99 except I60 to I69); c.) Digestive system (ICD10 codes: K00 to K93); d.) Neoplasms (ICD10 codes: C00 to D48); e.) Respiratory system (ICD10 codes: J00 to J99); and f.) Injury, poisoning and other external causes (ICD10 codes: S00 to T98). The analysis was performed using STATA 11 (Stata Corp., College Station, TX, USA) and Microsoft Excel 2010, (Redmond, WA, USA).

We also performed a systematic search in order to review the related literature investigating long-term survival amongst post-ICU patients in low and middle income countries. The search strategy and inclusion criteria are provided in Additional file 1.

## Results

**Figure 1:**
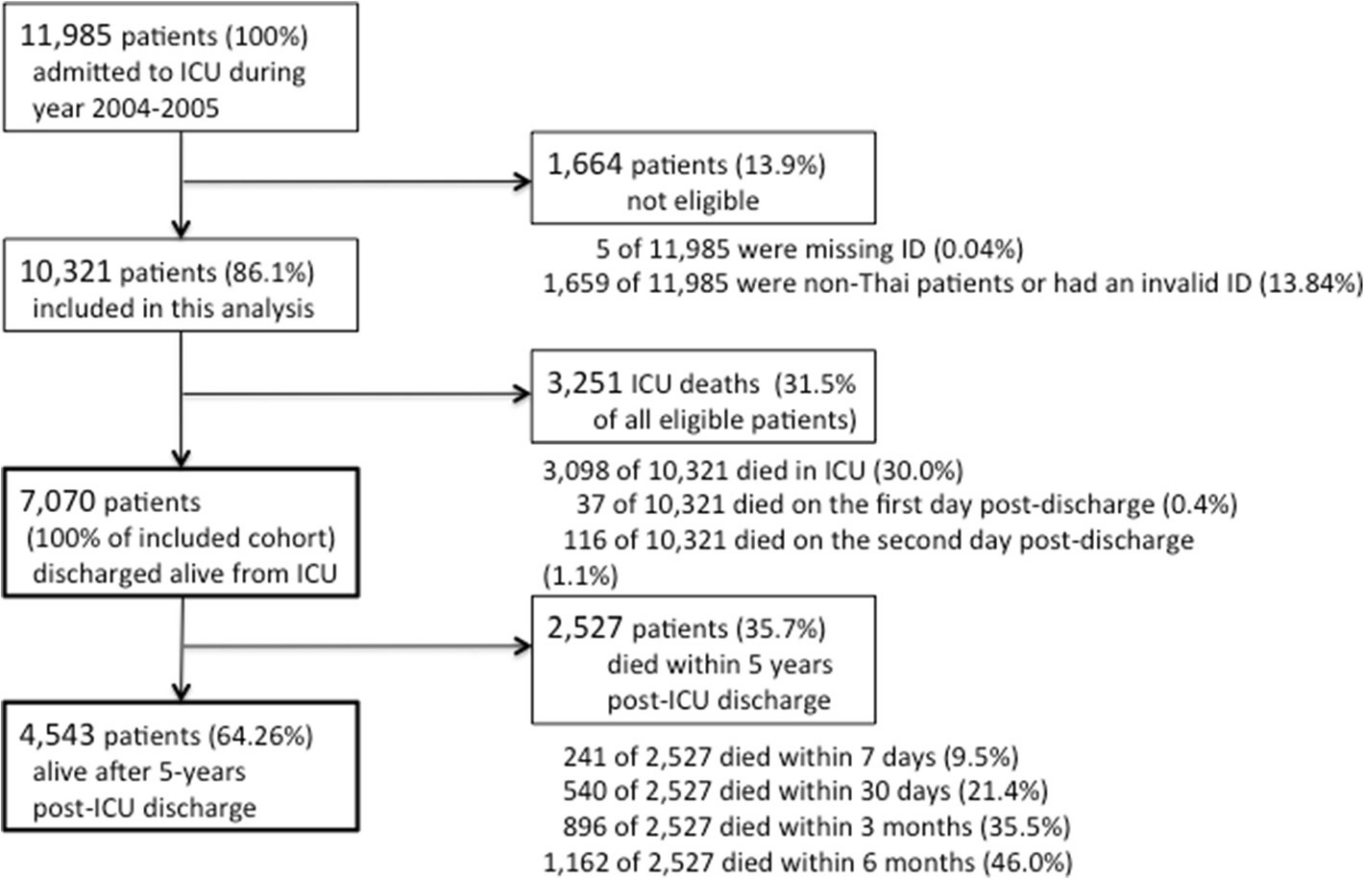
Patient flow from intensive care unit admission to discharge and until five-year follow-up.

**Figure 2:**
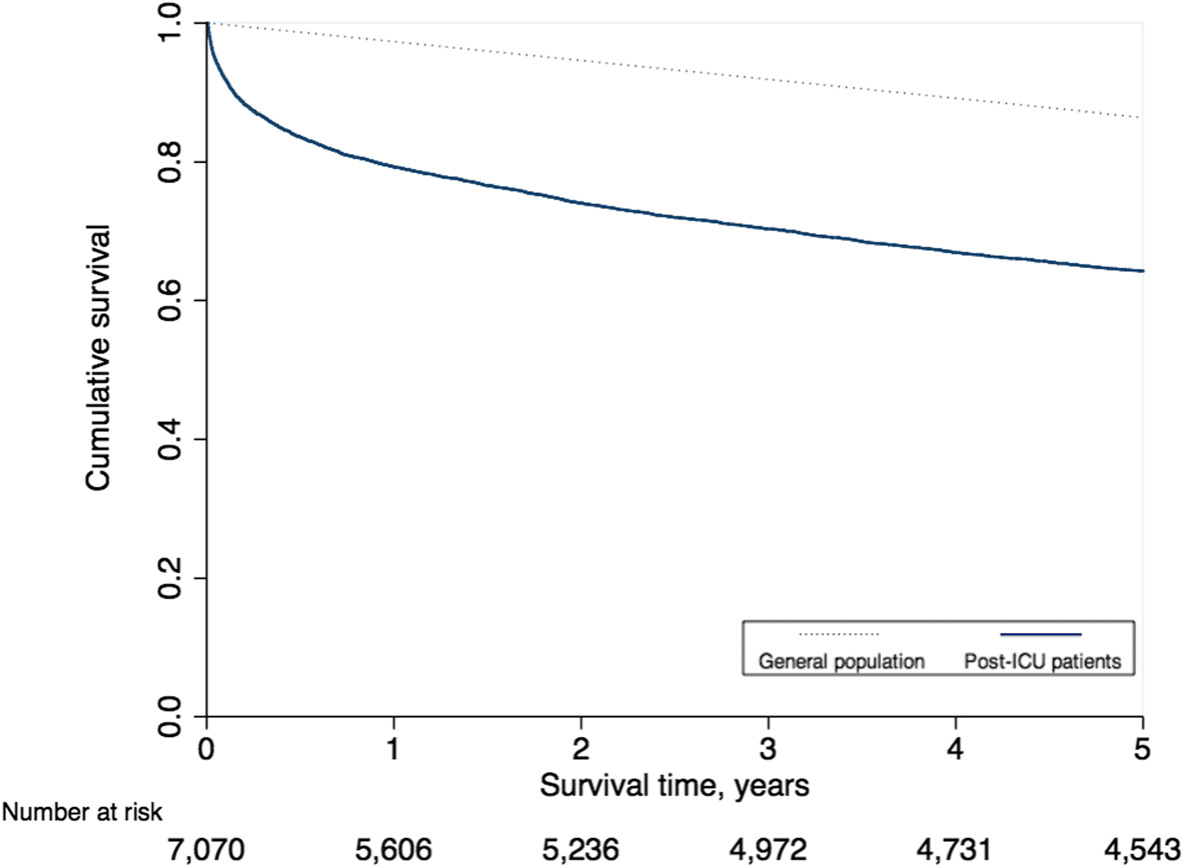
Kaplan-Meier survival estimates for post-ICU patients and survival for the general population in Thailand.

**Figure 3:**
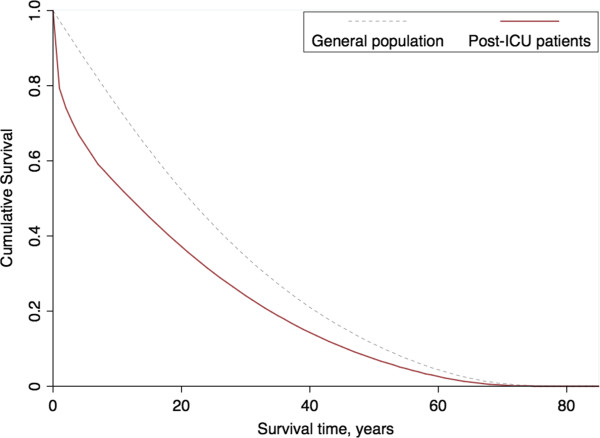
The extrapolated lifetime survival curve of post-ICU patients and survival for the general population.

There were 11,985 adult patients admitted to an ICU in Sappasithiprasong Hospital between 2004 and 2005 and discharged before 1 January 2006. After verifying the hospital dataset, 1,664 patients (13.9%) were not eligible for this analysis due to missing data, incomplete or invalid ID numbers, or coming from other countries (and therefore not recorded in the regional mortality records). As a result, 10,321 patients were included in this analysis. There were 7,223 patients who were discharged alive from the ICU; 153 of these died within two days and were counted as ICU deaths. Of these 61 (39.9%) died at the hospital and 92 (60.1%) died at home. We studied five-year survival in the remaining 7,070 patients who were discharged from the ICU alive (31.5% ICU fatality rate). Patient-flow is shown in Figure 1. Demographics and ICD10 codes in the group of patients who were discharged alive differed slightly from those in patients who died within the ICU (Table 1). In contrast, the group of post-ICU patients who died within five years of discharge tended to be older and much less likely to have ICD10 codes relating to injury, poison and other external causes than those who were alive after five years (Table 2). Of the 7,070 patients who were discharged alive, 79.3% survived the first year, then 74.0%, 70.3%, 66.9% and 64.2% survived each subsequent year (Figure 2). Overall, within five years, 2,527 of the original 7,070 (35.7%) had died. The Kaplan-Meier survival curve indicated a greatly elevated risk of death in the first year post-ICU discharge, with 9.5% (241 of 2,527) of all deaths occurring within seven days. Of these, 67 (27.8%) died at the hospital and 174 (72.2%) died at home. Of the total 2,527 deaths over five years, 21.4% (540) occurred within the first month, 35.5% (896) within three months, 46.0% (1,162) within six months, and 57.9% (1,464) within the first year. Mortality rates became close to those in the general population between years two to five after ICU discharge. The annual risks of death for each year during these periods were 0.21, 0.07, 0.05, 0.05 and 0.04, respectively. In the general population, the annual risk of death (matched for age and sex with those discharged alive from the ICU) was 0.03. Overall, half of the post-ICU patients would have been expected to die within 12.1 years of ICU discharge under base case assumptions. In a sample of the general population matched for age and sex, half would be expected to die within 21.2 years (Figure 3). The LE under base case assumptions and the sensitivity analysis are presented in Table 3. The overall LE amongst post-ICU patients was estimated to be 18.3 years while the LE in the general population (matched for age and sex) was estimated to be 25.2 years. The sensitivity analysis yielded estimates of LE 1.6% higher than under the base case assumption.

**Table 1:** Demographic data for ICU patients

	**Patients dying in the ICU**^**1**^	**Patients discharged alive from the ICU**
	**N = 3,251**	**N = 7,070**
Age (Med [IQR])	57.6 [42.6, 71.1]	54.5 [38.2, 67.8]
Age group (number of patients)		
15 to 29	408 (12.6%)	1,132 (16.1%)
30 to 44	492 (15.1%)	1,298 (18.4%)
45 to 59	879 (27.0%)	1,821 (25.8%)
60 to 74	901 (27.7%)	1,914 (27.1%)
>75	571 (17.6%)	905 (12.8%)
Length of hospital stay	3.0 [1,7]	7.0 [3,12]
(Med [IQR])		
Sex (% female)	1,241 (38.2%)	2,700 (38.2%)
ICD10 (Top five, by %)		
Circulatory system (I00 to I99)	1,040 (32.0%)	2,627 (37.2%)
- Cerebrovascular diseases (I60 to I69)	535	404
- Other forms of heart disease (I30 to I52)	179	615
- Ischemic heart diseases (I20 to I25)	152	886
- Chronic rheumatic heart diseases (I05 to I09)	86	441
Injury, poison and other external causes (S00 to T98)	751 (23.1%)	1,598 (22.6%)
- Injury (S00 to T14)	715	1,490
- Poisoning and certain other consequences of external causes (T15 to T98)	36	108
Digestive system (K00 to K93)	328 (10.1%)	789 (11.2%)
- Other diseases of the digestive system (K90 to K93)	106	125
- Diseases of oesophagus, stomach and duodenum (K20 to K31)	48	225
- Disorders of gallbladder, biliary tract and pancreas (K80 to K87)	43	166
Respiratory system (J00 to J99)	248 (7.6%)	376 (5.3%)
- Influenza and pneumonia (J09 to J18)	145	129
- Chronic lower respiratory diseases (J40 to J47)	35	82
- Suppurative and necrotic conditions of lower respiratory tract (J85 to J86)	10	61
Neoplasms (C00 to D48)	186 (5.7%)	488 (6.9%)
- Malignant neoplasms (C00 to C99)	160	331
- Neoplasms of uncertain or unknown behaviour (D37 to D48)	22	128
- Benign neoplasms (D10 to D36)	4	27
Hospital mortality (%)	N/A	139 (2.0%)
Five-year mortality (%)	N/A	2,527 (35.7%)

^1^Includes patients who died within two days of ICU discharge.

**Table 2:** Demographic data for post-ICU patients

	**Post-ICU patients dying within five years of ICU discharge**	**Post-ICU patients alive five years after ICU discharge**
	**N = 2,527**	**N = 4,543**
Age (Med [IQR])	64.6 [52.6, 74.0]	47.46 [32.6, 62.2]
Age group (number of patients)		
15 to 29	127 (5.0%)	1,005 (22.1%)
30 to 44	256 (10.1%)	1,042 (22.9%)
45 to 59	618 (24.5%)	1,203 (26.5%)
60 to 74	949 (37.6%)	965 (21.2%)
>75	577 (22.8%)	328 (7.2%)
Length of hospital stay	8.0 [4,15]	7.0 [3,11]
(Med [IQR])		
Sex (% female)	1,041 (41.2%)	1,659 (36.5%)
ICD10 (Top five, by %)		
Circulatory system (I00 to I99)	1,023 (40.5%)	1,604 (35.3%)
- Cerebrovascular diseases (I60 to I69)	337	549
- Other forms of heart disease (I30 to I52)	225	390
- Ischemic heart diseases (I20 to I25)	216	188
- Chronic rheumatic heart diseases (I05 to I09)	114	327
Neoplasms (C00 to D48)	324 (12.8%)	164 (3.6%)
- Malignant neoplasms (C00 to C99)	249	82
- Neoplasms of uncertain or unknown behaviour (D37 to D48)	70	58
- Benign neoplasms (D10 to D36)	5	22
Digestive system (K00 to K93)	321 (12.7%)	468 (10.3%)
- Other diseases of the digestive system (K90 to K93)	86	139
- Diseases of oesophagus, stomach and duodenum (K20 to K31)	67	99
- Disorders of gallbladder, biliary tract and pancreas (K80 to K87)	60	65
Injury, poison and other external causes (S00 to T98)	215 (8.5%)	1,383 (30.4%)
- Injury (S00 to T14)	182	1,308
- Poisoning and certain other consequences	33	75
of external causes (T15 to T98)		
Respiratory system (J00 to J99)	190 (7.5%)	186 (4.1%)
- Influenza and pneumonia (J09 to J18)	75	54
- Chronic lower respiratory diseases (J40 to J47)	58	46
- Suppurative and necrotic conditions	15	24
of lower respiratory tract (J85 to J86)		

**Table 3:** Life expectancy among post-ICU patients adjusted for age and sex

**Age group (Years)**		** Life expectancy (LE)**	
	**†Base case**	*Sensitivity analysis	**◊General population**
15 to 29	43.16	43.80	48.97
30 to 44	28.87	29.56	36.01
45 to 59	16.41	16.98	24.03
60 to 74	8.72	8.96	13.66
≥75	4.75	4.61	6.36
Overall	18.26	18.56	25.15

†Base case analysis: we assumed that from year five to year seven post-discharge the relative risk of mortality amongst post-ICU patients was the same as that in year five. From year eight onwards, the relative risk was assumed to be 1.

*Sensitivity analysis: we assumed the mortality rates among the post-ICU patients after five years post-discharge to be the same as those in the general population matched for age and sex.

◊General population: we applied the mortality rate of the Thai general population age- and sex-matched to all post-ICU patients since ICU discharge.

**Figure 4:**
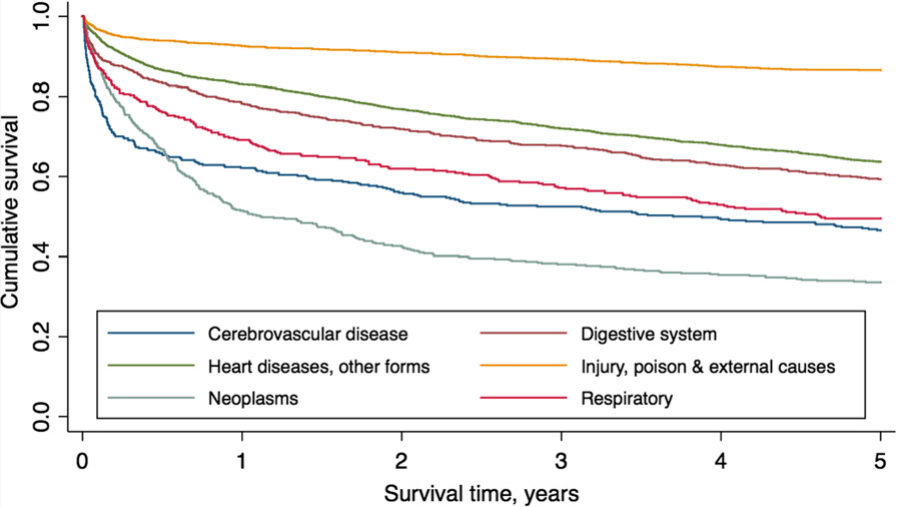
Kaplan-Meier survival estimates for post-ICU patients stratified by diagnostic group.

Survival categorised by specific diagnostic categories is presented in Figure 4 and Table 4. The lowest survival within six months of discharge was seen in patients admitted with cerebrovascular disease, though at five years post-discharge the lowest survival (33.6%) was seen in patients with neoplasms. The highest survival rates were consistently seen in those admitted due to injury, poisoning or other external causes; 86.5% of patients in this group survived at least five years.

**Table 4:** Comparison of survival 1-5 years after ICU discharged by age and diagnostic group

**Time of follow-up**	**At ICU discharge**	**1 year**	**2 year**	**3 year**	**4 year**	**5 year**
Number of patients at each follow up time	7,070	5,606	5,236	4,972	4,730	4,543
Age (median, [IQR])	54.5 [38.2, 67.8]	51.4 [35.2, 65.6]	50.5 [34.2, 64.8]	49.5 [33.8, 63.8]	48.5 [33.0, 63.0]	47.5 [32.6, 62.2]
Length of hospital stay	7.0 [3,12]	7.0 [3,11]	7.0 [3,11]	7.0 [3,11]	7.0 [3,11]	7.0 [3,11]
Sex (% female)	38.2%	37.6%	37.3%	37.2%	36.7%	36.5%
Age group						
15 to 29	100.0%	93.0%	91.8%	90.7%	89.8%	88.8%
30 to 44	100.0%	88.4%	85.1%	83.1%	81.8%	80.3%
45 to 59	100.0%	80.2%	75.0%	71.4%	68.4%	66.1%
60 to 74	100.0%	70.7%	64.5%	58.9%	53.7%	50.4%
≥75	100.0%	65.4%	54.6%	48.5%	41.9%	36.2%
Diagnostic group						
Heart diseases, other forms (I00 to I99 except I60 to I69)	100.0%	83.0%	76.9%	72.0%	67.9%	63.7%
Cerebrovascular diseases (I60 to I69)	100.0%	62.1%	55.9%	52.5%	49.3%	46.5%
Injury, poison and other external causes (S00 to T98)	100.0%	92.6%	90.9%	89.3%	87.4%	86.5%
Digestive system (K00 to K93)	100.0%	78.2%	71.9%	67.7%	62.9%	59.3%
Respiratory system (J00 to J99)	100.0%	69.1%	62.0%	57.2%	52.9%	43.6%
Neoplasms (C00 to D48)	100.0%	51.4%	42.4%	38.1%	35.5%	33.6%

In the systematic search for studies of long-term survival amongst post-ICU patients in low and middle income countries, we found only one study evaluating post-ICU survival across all diagnostic categories [26]. This study followed up 187 post-ICU patients in Malaysia for two years. It was reported that 97 of 105 post-ICU patients (92.4%) who responded to a questionnaire survived for two years. However, the high loss to follow-up in this study (43.8%, 82 from 187) makes interpretation of these findings difficult.

## Discussion

This study found that post-ICU patients had a substantially higher mortality rate (and substantially reduced LE) compared to the general population, with most of the difference seen in the first year post-discharge. Overall, the LE among the post-ICU patients was estimated to be seven years lower than in the general population and the number of life years gained from preventing one ICU death was found to be about two-thirds that from preventing one death in the general population (matched for age and sex).

Results from this study are broadly consistent with those from previous studies conducted elsewhere in high-income countries [2,13,14,16-18,36-38]. Our estimate that cumulative mortality over the five years was 35.7% (or 2.5 times higher than in an age- and sex-matched general population) is slightly higher but comparable with estimates from previous studies which found that the five years cumulative mortality rate ranged from 17.9 to 33.5% [2,13,14,16-18,36-38]. Our estimate of the risk of death in year five, 0.04, is at the upper end of the range estimated in studies conducted in high-income countries (0.01 to 0.04) [14,36,37]. The mortality rates among post-ICU patients in our study were high in the first 12 months, then decreased rapidly, and were projected to closely approximate those of the general population by year eight post-ICU discharge. Studies conducted in Finland, Norway and Scotland [13,36,37] also demonstrated substantially greater risk of death during the first year, but these became similar to the general population within one to four years. On the other hand, studies conducted in the United Kingdom [16] and Australia [38] found that the mortality rate amongst former ICU patients was higher than the general population over a 5-year and 15-year follow-up period, respectively.

There are several possible reasons for differences in the time for post-ICU mortality rates to approach those in the general population. Firstly, there was considerable variation between the studies in the frequency of different diagnostic categories. Our study had a relatively high proportion of patients with ICD10 codes relating to injury, poisoning and other external causes (23% compared to a range of 7 to 15% in other studies) [18,36-38]. Conversely, there was a low proportion of patients with ICD10 codes relating to the respiratory system (5% compared with a range of 8 to 36%) [13,36,37]. Figure 4 suggests that these differences are likely to be associated with both a shorter period for post-ICU mortality to approach that in the general population and a relatively high five-year post-ICU survival rate.

Quality of care in different settings [39,40] is another possible factor that could impact on long-term survival rates. Higher quality of care should reduce ICU mortality, but could potentially either increase or decrease the long-term survival in patients discharged alive from ICU. The latter could occur if higher quality of care prevents ICU deaths in patients with poor long-term prognosis (where some of these patients would have died in the ICU if in lower quality of care settings). Quantifying such competing effects is challenging, but important for evaluating the cost-effectiveness of interventions to improve quality of ICU care in low and middle income countries.

Currently, however, there are few studies of long-term survival following ICU stays in lower and middle income countries. While the systematic search identified a small number of studies evaluating long-term survival following ICU discharge in specific diagnostic categories (liver transplants, myocardial infarction, metastatic solid cancer, chronic obstructive pulmonary disease) [41-45], long-term follow-up of representative ICU cohorts was lacking.

Our analysis accounted for the common practice in Southeast Asia of discharging moribund patients to die at home by classifying deaths occurring within two days of discharge as ICU deaths. The two-day cut-off was chosen because post-ICU mortality showed a clear spike on day two post-discharge (with 116 deaths, or 1.12% of total ICU patients) but showed a gradual decline from day three (48 (0.47%), 46 (0.45%), 36 (0.35%), 28 (0.27%) and 29 (0.28%) for days three to seven, respectively). This resulted in only slightly higher ICU mortality than would have been obtained had we only considered deaths occurring during the admission (31.5% versus 30.0% mortality, or 153 more deaths), and consequently, slightly lower cumulative five-year mortality amongst the non-ICU deaths (37.1% versus 35.7%).

The mortality rates during years six and seven post-ICU discharge are likely to be somewhat lower than assumed in the base case (which assumed the same relative risk for death as in year five), but somewhat higher than assumed in the sensitivity analysis (which assumed a relative risk of one). However, these two assumptions yielded estimates of LE that differed by less than 2% (Table 3) indicating that improved estimates of mortality in years six and seven would have negligible impact on the results.

Interestingly, among individuals over 75 years of age, the mortality rate was higher in the post-ICU group than in the general population in the first two years, but lower in the following years, resulting in a slightly longer LE than the general population. This might be explained by the possibility that these patients are on average healthier than the general population, having survived their ICU admission.

### Limitations

This study has several limitations. Data from a single regional hospital may not be representative of the national population due to differences in patient characteristics and quality of hospital care. However, similar regional hospitals provide care to most of the population in Thailand and the large population (n >7,000) and long-term follow-up strengthen our findings. Nonetheless, had resources permitted, this study could have been improved (and its external validity strengthened) by collecting data from multiple sites across Thailand. A second limitation is that this study was based on retrospective data, which were inevitably incomplete. Moreover, as the regional death registry was used (not national data), it is possible that we have missed some deaths in patients who moved and died outside of the northeast region. Our analysis might, therefore, underestimate mortality. However, any such bias is likely to be small as the five-year migration rate amongst the northeast Thai population was estimated to be 3.1% in 2000 [46]. This rate is likely to be even lower in older age groups where most of the mortality occurs. Another limitation is the lack of a standardised measure of severity of illness. A standard severity score (such as Acute Physiology and Chronic Health Evaluation (APACHE) II) would have helped to inform comparisons of our findings with those from other studies, but such data are not routinely collected in ICUs in Thailand. Finally, this study would have been improved by the addition of Health Related Quality of Life (HRQOL) data to estimate the quality adjusted life expectancy (QALE) amongst the post-ICU patients. Ideally, such HRQOL information would be obtained from a long-term cohort study in the local population; resources for this were not available to us. Given the range of the HRQOL between 0.56 and 0.88 as shown in the literature [13,14,16,19] (all from high-income countries) the expected QALE of post-ICU patients would range from 10.2 to 16.1 QALYs. Prospective collection of such quality of life data is an important area for future health economic research in developing countries.

## Conclusions

This study represents one of the first attempts to estimate long-term post-ICU survival in a developing country context. Post-ICU patients had higher mortality than members of the general population (matched for age and sex) over the five-year follow-up period. The estimated LE is useful for economic evaluations and should support decision-makers considering potential investments in interventions that could prevent unnecessary deaths during ICU or hospital admissions.

### Key messages

•Five-year mortality amongst post-ICU patients in Thailand was estimated to be 35.7%. This is about 2.5 times higher than that in the general population (age and sex matched).

•The risk of death was greatly elevated in the first year after ICU discharge and approached that in the general population in subsequent years.

•The extrapolated lifetime survival indicated that post-ICU patients had 27.4% lower life expectancy than the general population (age and sex matched).

•Patients admitted to the ICU as a result of injury, poisoning or other external causes had the lowest mortality rate over the five-year follow-up; patients with neoplasms had the highest.

•Estimates of the number of life years gained from interventions preventing ICU deaths will aid policy-makers considering potential investments in this area.

## Abbreviations

DALY: Disability adjusted life year; HRQOL: Health-related quality of life; ICU: Intensive care unit; ID: Identification number; LE: Life expectancy; LY: Life year; QALE: Quality adjusted life expectancy; QALY: Quality adjusted life year.

## Competing interests

The authors declare that they have no competing interests.

## Authors’ contributions

NL, BSC, YL, DL, NG and ND contributed to the study conception and design. MH, DL, SC and PT collected the data. NL and MH performed the data analysis. NL, BSC and YL wrote the draft. DL, MH, SC, PT, NG and ND critically revised the manuscript for important intellectual content. All authors read and approved the final manuscript.

## Supplementary Material

Additional file 1 Three main phrases used in the systematic literature search. Click here for file
