# Supplementary material for: Long-term survival after intensive care unit discharge in Thailand: a retrospective study
Source: Crit Care. 2013 Oct 3;17(5):R219. doi: 10.1186/cc13036 (PMC4056652; doi:10.1186/cc13036)
Supplement: Additional file 1 — Three main phrases used in the systematic literature search. [file cc13036-S1.doc]

**Three main phrases used in the systematic literature search**

1. Intensive care unit (ICU)

2. Long term follow-up

3. Low and middle income countries

Keywords:

(“intensive care unit” or “critical care unit” or “intensive therapy unit” or “intensive treatment unit”) and (“post-discharge” or “post discharge” or “post-ICU discharge” or “post ICU discharge” or “after discharge” or “long-term outcome” or “long term outcome” or “long-term follow up” or “long term follow up” or “long-term survival” or “long term survival” or “five-year survival” or “five year survival” or “five years survival” or “five years” or “two-year survival” or “two year survival” or “one-year survival” or “one year survival”) and (“low income“ or “developing country” or “middle income” or “low-middle” or “upper-middle-income” or “lower-middle-income” or “Afghanistan” or “Gambia” or “Mozambique” or “Bangladesh” or “Guinea“ or “Myanmar” or “Benin” or “Guinea-Bisau” or “Nepal” or “Burkina Faso” or “Haiti” or “Niger” or “Burundi” or “Kenya” or “Rwanda” or “Cambodia” or “Korea, Dem Rep” or “Republic of Korea” or “Sierra Leone” or “Central African Republic” or “Kyrgyz Republic” or “Somalia” or “Chad” or “Liberia” or “Tajikistan” or “Comoros” or “Madagascar” or “Tanzania” or “Congo, Dem. Rep” or “Congo” or “Malawi” or “Togo” or “Eritrea” or “Mali” or “Uganda” or “Ethiopia” or “Mauritania” or “Zimbabwe” or “Albania” or “Indonesia” or “Samoa” or “Armenia” or “India” or “São Tomé and Principe” or “Belize” or “Iraq” or “Senegal” or “Bhutan” or “Kiribati” or “Solomon Islands” or “Bolivia” or “Kosovo” or “South Sudan” or “Cameroon” or “Lao” or “Sri Lanka” or “Cape Verde” or “Lesotho” or “Sudan” or “Congo, Rep.” or “Marshall Islands” or “Swaziland” or “Côte d’Ivoire” or “Micronesia” or “Syrian” or “Djibouti” or “Moldova” or “Timor-Leste” or “Egypt” or “Mongolia” or “Tonga” or “El Salvador” or “Morocco” or “Ukraine” or “Fiji” or “Nicaragua” or “Uzbekistan” or “Georgia” or “Nigeria” or “Vanuatu” or “Ghana” or “Pakistan” or “Vietnam” or “Guatemala” or “Papua New Guinea” or “West Bank and Gaza” or “Guyana” or “Paraguay” or “Yemen” or “Honduras” or “Philippines” or “Zambia” or “Angola” or “Ecuador” or “Palau” or “Algeria” or “Gabon” or “Panama” or “American Samoa” or “Grenada” or “Peru” or “Antigua and Barbuda” or “Iran” or “Romania” or “Argentina” or “Jamaica” or “Russia” or “Azerbaijan” or “Jordan” or “Serbia” or “Belarus” or “Kazakhstan” or “Seychelles” or “Bosnia and Herzegovina” or “Latvia” or “South Africa” or “Botswana” or “Lebanon” or “St. Lucia” or “Brazil” or “Libya” or “St. Vincent and the Grenadines” or “Bulgaria” or “Lithuania” or “Suriname” or “Chile” or “Macedonia” or “Thailand” or “China” or “Malaysia” or “Tunisia” or “Colombia” or “Maldives” or “Turkey” or “Costa Rica” or “Mauritius” or “Turkmenistan” or “Cuba” or “Mexico” or “Tuvalu” or “Dominica” or “Montenegro” or “Uruguay” or “Dominican Republic” or “Namibia” or “Venezuela”)

Pubmed search:

((“intensive care units”[MeSH Terms] OR (“intensive”[All Fields] AND “care”[All Fields] AND “units”[All Fields]) OR “intensive care units”[All Fields] OR (“intensive”[All Fields] AND “care”[All Fields] AND “unit”[All Fields]) OR “intensive care unit”[All Fields]) OR (“intensive care units”[MeSH Terms] OR (“intensive”[All Fields] AND “care”[All Fields] AND “units”[All Fields]) OR “intensive care units”[All Fields] OR (“critical”[All Fields] AND “care”[All Fields] AND “unit”[All Fields]) OR “critical care unit”[All Fields]) OR (intensive[All Fields] AND (“therapy”[Subheading] OR “therapy”[All Fields] OR “therapeutics”[MeSH Terms] OR “therapeutics”[All Fields]) AND unit[All Fields]) OR (intensive[All Fields] AND (“therapy”[Subheading] OR “therapy”[All Fields] OR “treatment”[All Fields] OR “therapeutics”[MeSH Terms] OR “therapeutics”[All Fields]) AND unit[All Fields]))

AND

(“post-discharge”[All Fields] OR “post discharge”[All Fields] OR “post-ICU discharge”[All Fields] OR “post ICU discharge”[All Fields] OR “after discharge”[All Fields] OR “long-term outcome”[All Fields] OR “long term outcome”[All Fields] OR “long-term follow up”[All Fields] OR “long term follow up”[All Fields] OR “long-term survival”[All Fields] OR “long term survival”[All Fields] OR “longterm survival”[All Fields] OR “five-year survival”[All Fields] OR “five year survival”[All Fields] OR “five years survival”[All Fields] OR “five years”[All Fields] OR “two-year survival”[All Fields] OR “two year survival”[All Fields] OR “one-year survival”[All Fields] OR “one year survival”[All Fields])

AND

(“low income”[all fields] or “developing country”[all fields] or “middle income”[all fields] or “low-middle”[all fields] or “upper-middle-income”[all fields] or “lower-middle-income”[all fields] or “afghanistan”[all fields] or “gambia”[all fields] or “mozambique”[all fields] or “bangladesh”[all fields] or “guinea”[all fields] or “myanmar”[all fields] or “benin”[all fields] or “guinea-bisau”[all fields] or “nepal”[all fields] or “burkina faso”[all fields] or “haiti”[all fields] or “niger”[all fields] or “burundi”[all fields] or “kenya”[all fields] or “rwanda”[all fields] or “cambodia”[all fields] or “korea dem rep”[all fields] or “republic of korea”[all fields] or “sierra leone”[all fields] or “central african republic”[all fields] or “kyrgyz republic”[all fields] or “somalia”[all fields] or “chad”[all fields] or “liberia”[all fields] or “tajikistan”[all fields] or “comoros”[all fields] or “madagascar”[all fields] or “tanzania”[all fields] or (“congo, dem. rep”[all fields]) or “malawi”[all fields] or “togo”[all fields] or “eritrea”[all fields] or “mali”[all fields] or “uganda”[all fields] or “ethiopia”[all fields] or “mauritania”[all fields] or “zimbabwe”[all fields] or “albania”[all fields] or “indonesia”[all fields] or “samoa”[all fields] or “armenia”[all fields] or “india”[all fields] or “sao tome and principe”[all fields] or “belize”[all fields] or “iraq”[all fields] or “senegal”[all fields] or “bhutan”[all fields] or “kiribati”[all fields] or “solomon islands”[all fields] or “bolivia”[all fields] or “kosovo”[all fields] or “south sudan”[all fields] or “cameroon”[all fields] or “lao”[all fields] or “sri lanka”[all fields] or “cape verde”[all fields] or “lesotho”[all fields] or “sudan”[all fields] or “congo, rep.”[all fields] or “marshall islands”[all fields] or “swaziland”[all fields] or “cote d’ivoire”[all fields] or “micronesia”[all fields] or “syrian”[all fields] or “djibouti”[all fields] or “moldova”[all fields] or “timor-leste”[all fields] or “egypt”[all fields] or “mongolia”[all fields] or “tonga”[all fields] or “el salvador”[all fields] or “morocco”[all fields] or “ukraine”[all fields] or “fiji”[all fields] or “nicaragua”[all fields] or “uzbekistan”[all fields] or “georgia”[all fields] or “nigeria”[all fields] or “vanuatu”[all fields] or “ghana”[all fields] or “pakistan”[all fields] or “vietnam”[all fields] or “guatemala”[all fields] or “papua new guinea”[all fields] or “west bank and gaza”[all fields] or “guyana”[all fields] or “paraguay”[all fields] or “yemen”[all fields] or “honduras”[all fields] or “philippines”[all fields] or “zambia”[all fields] or “angola”[all fields] or “ecuador”[all fields] or “palau”[all fields] or “algeria”[all fields] or “gabon”[all fields] or “panama”[all fields] or “american samoa”[all fields] or “grenada”[all fields] or “peru”[all fields] or “antigua and barbuda”[all fields] or “iran”[all fields] or “romania”[all fields] or “argentina”[all fields] or “jamaica”[all fields] or “russia”[all fields] or “azerbaijan”[all fields] or “jordan”[all fields] or “serbia”[all fields] or “belarus”[all fields] or “kazakhstan”[all fields] or “seychelles”[all fields] or “bosnia and herzegovina”[all fields] or “latvia”[all fields] or “south africa”[all fields] or “botswana”[all fields] or “lebanon”[all fields] or “st. lucia”[all fields] or “brazil”[all fields] or “libya”[all fields] or “st. vincent and the grenadines”[all fields] or “bulgaria”[all fields] or “lithuania”[all fields] or “suriname”[all fields] or “chile”[all fields] or “macedonia”[all fields] or “thailand”[all fields] or “china”[all fields] or “malaysia”[all fields] or “tunisia”[all fields] or “colombia”[all fields] or “maldives”[all fields] or “turkey”[all fields] or “costa rica”[all fields] or “mauritius”[all fields] or “turkmenistan”[all fields] or “cuba”[all fields] or “mexico”[all fields] or “tuvalu”[all fields] or “dominica”[all fields] or “montenegro”[all fields] or “uruguay”[all fields] or “dominican republic”[all fields] or “namibia”[all fields] or “venezuela”[all fields])

Inclusion criteria:

| a) | Populations are the patients who had been admitted to adults intensive care units (ICUs) including specialised ICUs; medicine/coronary care ICUs, surgical/neurosurgical ICUs, trauma ICUs and cardiothoracic ICU. Studies confined to certain underlying or specific diseases such as sepsis, transplantations and cardiac surgery will be excluded. |
| --- | --- |
| b) | Follow-up period must be at least one year from ICU/hospital admission or ICU/hospital discharge. |
| c) | No restrictions regarding language or year of publication. |
